# Supplementary material for: Rotation quenches in trapped bosonic systems
Source: Sci Rep. 2025 Jul 26;15:27193. doi: 10.1038/s41598-025-07144-w (PMC12297281; doi:10.1038/s41598-025-07144-w)
Supplement: Supplementary file 1 — Supplementary Information. [file 41598_2025_7144_MOESM1_ESM.zip › Revised_41598_2025_7144_MOESM1_ESM/Supplementary_material/Supplementary_Material.pdf]

Rotation quenches in trapped bosonic systems
(Supplementary Material)

Rhombik Roy,[1, 2, *] Sunayana Dutta,[1, 2] and Ofir E. Alon[1, 2]

[1] *Department of Physics, University of Haifa, Haifa 3498838, Israel*

[2] *Haifa Research Center for Theoretical Physics and Astrophysics, University of Haifa, Haifa 3498838, Israel*

This supplementary material offers an in-depth many-body analysis of the main findings. Section I outlines the procedure for quantifying and categorizing the quench magnitudes. We differentiate the small, intermediate, and large rotation quenches by evaluating the ground-state energy difference between the initial and the quenched states. The many-body analysis is performed for $N = 8$ bosons interacting with repulsive Gaussian interaction potential, with the interaction parameter of $\Lambda = \lambda_0(N-1) = 0.1$. Our numerical calculations employ the multiconfigurational time-dependent Hartree method for bosons (MCTDHB) [1–3], available in the multiconfigurational time-dependent Hartree method for indistinguishable particles software [4, 5]. Known for its high accuracy, MCTDHB can effectively solve the many-body Schrödinger equation across both inertial [6–8] and non-inertial reference frames [9]. We solve the dimensionless Schrödinger equation by converting the Hamiltonian into a dimensionless form. This is done by dividing the original Hamiltonian by $\frac{\hbar^2}{mL^2}$, where L is a convenient length scale and m is the mass of the boson. The main text examines both mean-field and many-body dynamics. For the mean-field analysis, we set $M = 1$ in the MCTDHB calculations. In this regime, the dynamics are controlled only by the mean-field interaction parameter Λ. Thus, in the mean-field study, we can increase N to any number while maintaining a fixed Λ value.

Sec. II demonstrates the convergence of our results by analyzing the number of time-adaptive orbitals and the variance of several observables. Sec. II B 1 demonstrates the convergence of many-body results in the symmetric trap. Sec. II B 2 presents the convergence of many-body calculations in the elongated trap potential. Sec. II B 3 discusses the convergence of many-body calculations in the four-fold symmetric trap potential, only for the small and the intermediate rotation quenches. Due to the participation of numerous excited states in the dynamics after the large rotation quench in the four-fold symmetric trap, achieving convergence becomes extremely challenging. Detailed dynamical measures and their convergences for the large rotation quench is presented in Sec. II C, considering $N = 4$ bosons and a higher number of orbitals. We have also performed the convergence tests with respect to spatial grid points (not presented here). While a 64×64 grid points are sufficient to accurately capture the dynamical evolution, we employed a higher-resolution of 128×128 grid points

* rroy@campus.haifa.ac.il

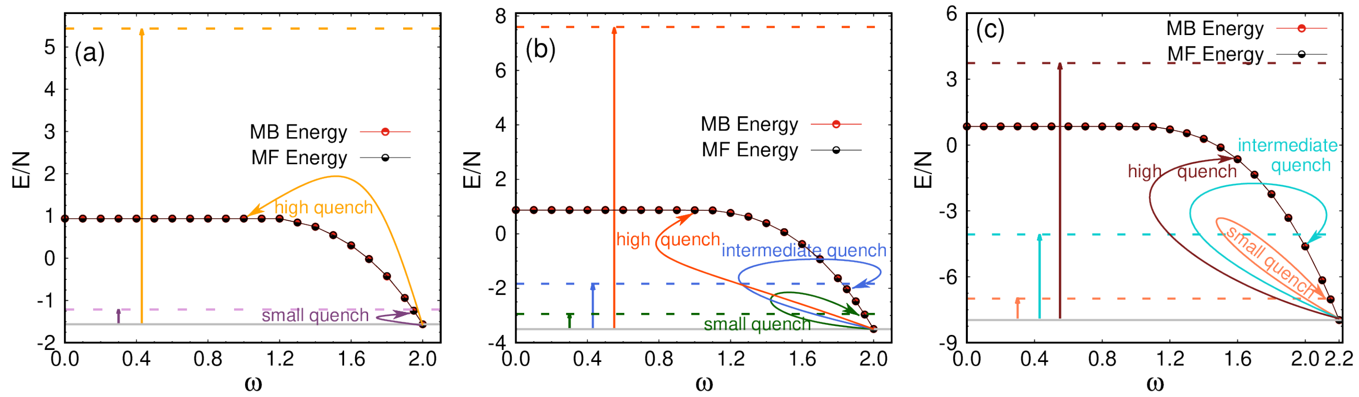

FIG. 1: **Energy diagram and the amount of energy pumped into the system.** The ground-state energy per particle is shown as a function of the rotation frequency in (a) the symmetric trap; (b) the elongated trap; and (c) the four-fold symmetric trap. Both mean-field and many-body energies fall on top of each other. The dotted lines corresponds to the energy per particle after the quench and the vertical arrows show the amount of energy pumped into the system during the quenches. Color codes are described in each panel. This plot offers insights into characterizing the strength of rotational quenches. All quantities shown are dimensionless.

to enhance the visualization of the density distributions.

I. CHARACTERIZATION OF SMALL, INTERMEDIATE AND LARGE QUENCHES

This section outlines the selection process for the strength of rotation quenches. We calculate the ground-state energy for the bosons trapped in three different potentials. Fig. 1 shows how the ground-state energy per particle changes with different rotation frequencies. We present results for three trap configurations: (i) the symmetric trap, (ii) the elongated trap, and (iii) the four-fold symmetric trap. Our calculations are conducted at both mean-field and many-body levels. The ground-state energy calculations in both mean-field and many-body levels are exactly overlapped for all the cases. The strength of the rotation quench is determined by the ground-state energy difference between the initial and final states.

In the symmetric trap, the ground-state energy for the initial state prepared with $\omega_i = 2.0$ is $\frac{E}{N} = -1.56$. The effect of a rotation quench is analyzed for two final rotation frequencies: (i) $\omega_f = 1.95$ and (ii) $\omega_f = 1.0$. The ground-state energy per particle is $\frac{E}{N} = -1.23$ for $\omega = 1.95$ and $\frac{E}{N} = 0.93$ for $\omega = 1.0$. As a result, the energy differences relative to the initial state are $\Delta E = 0.33$ and $\Delta E = 2.49$, respectively. Based on the differences in ground-state energy, we classify the $\omega_i = 2.0 \rightarrow \omega_f = 1.95$ quench as the small rotation quench, as only a small amount of energy is injected into the system. The $\omega_i = 2.0 \rightarrow \omega_f = 1.0$ quench is labeled as the large rotation

quench, given the substantial energy pumped into the system.

After the quench, multiple excited states contribute to the dynamics, and the average energy levels after each quench are shown as a dotted line in Fig. 1(a). For the small rotation quench, the quenched energy level is slightly elevated at −1.21 ($E_g = -1.23$). The quenched energy level is uplifted significantly to 5.43 ($E_g = 0.93$) for the large rotation quench, which is sufficiently higher than the ground state.

Similarly, in the elongated trap, the initial state prepared with $\omega_i = 2.0$ has the energy per particle of $\frac{E}{N} = -3.49$. We examine the impact of the rotation quench for three final rotation frequencies: (i) $\omega_f = 1.95$, (ii) $\omega_f = 1.85$, and (iii) $\omega_f = 1.0$. The ground-state energy per particle is $\frac{E}{N} = -2.96$ for $\omega = 1.95$, $\frac{E}{N} = -2.03$ for $\omega = 1.85$, and $\frac{E}{N} = 0.86$ for $\omega = 1.0$. Consequently, the ground-state energy differences with respect to the initial state for the three quenches are $\Delta E = 0.53$, $\Delta E = 1.46$, and $\Delta E = 4.35$, respectively. Based on the ground-state energy differences, we categorize these quenches as small, intermediate, and large rotation quenches, as shown in Fig. 1(b).

After the quench, the average energy levels (dotted line in Fig. 1(b)) differ from their respective ground-state energies due to the participation of several excited states in the time evolution. For the small rotation quench, the quenched energy level is slightly elevated at −2.94 ($E_g = -2.96$). In intermediate rotation quench, the quenched energy level shows a comparatively higher value of −1.83 ($E_g = -2.03$). For the large rotation quench, the quenched energy level significantly rises to 7.59 ($E_g = 0.86$). In this case, the quenched state energy deviates significantly from the ground state, indicating substantial involvement of excited states, which necessitates a large Hilbert space for numerical convergence. The detailed convergence analysis are provided in later sections.

In the four-fold symmetric trap, we initialize the system with $\omega_i = 2.2$ and the energy per particle of the ground state is $\frac{E}{N} = -7.96$. We investigate the rotation quench dynamics for three different final frequencies: (i) $\omega_f = 2.15$, (ii) $\omega_f = 2.0$, and (iii) $\omega_f = 1.6$. The ground-state energy per particle is $\frac{E}{N} = -7.02$ for $\omega = 2.15$, $\frac{E}{N} = -4.61$ for $\omega = 2.0$, and $\frac{E}{N} = -0.64$ for $\omega = 1.6$. Therefore, the ground-state energy differences with respect to the initial state are $\Delta E = 0.94$, $\Delta E = 3.35$, and $\Delta E = 7.32$, respectively. We categorize these quenches as small, intermediate, and large based on the ground-state energy differences, as presented in Fig. 1(c).

After each quench, the average energy levels are shown as a dotted line in Fig. 1(c) which is higher than their respective ground states. The quenched energy level is slightly higher at −6.99 ($E_g = -7.02$) for the small rotation quench. In the intermediate rotation quench, the quenched energy level exhibits a comparatively higher value of −4.06 ($E_g = -4.61$). For the large rotation quench, the quenched energy level significantly increases to 3.73 ($E_g = -0.64$). In this case, obviously, numerous excited states are also involved in the dynamics. The four-fold

symmetric trapping potential makes it particularly difficult to achieve converged results, especially for the large rotation quench. As a trick, we reduce the number of particles to $N = 4$ (only for the large rotation quench) so that we can get the converged many-body results. The detailed discussion of the dynamics and the convergence for the large rotation quench in the four-fold symmetric trap is discussed in sec. II C.

It is important to note that we can also estimate how strong the quench is by looking at the number of vortices in the ground state of the initial and the final rotation frequency. Since the system is prepared with high rotation and quenched to a lower value, the quench therefore triggers a non trivial vortex dynamics in order to accomodate to the new stable conditions with a diminished number of vortices. Therefore, the greater the difference in the number of vortices, the more intense the resulting dynamics will be.

**Number of Vortices in the elongated trap**				
	Initial state ($\omega = 2.0$)	$\omega = 1.95$	$\omega = 1.85$	$\omega = 1.0$
Ground state	12	10 (-2)	8 (-4)	0 (-12)
During dynamics	—	$\sim 10-12$ (Small rotation quench)	$\sim 10-14$ (Intermediate rotation quench)	$\sim 12-16$ (Large rotation quench)

**Number of Vortices in the four-fold symmetric trap**				
	Initial state ($\omega = 2.2$)	$\omega = 2.15$	$\omega = 2.0$	$\omega = 1.6$
Ground state	24	24 (0,spreaded)	20 (-4)	12 (-12)
During dynamics	—	$\sim 20-24$ (Small rotation quench)	$\sim 24-28$ (Intermediate rotation quench)	$\sim 24-32$ (Large rotation quench)

TABLE I: The upper row presents the number of vortices in the ground state for different rotation frequencies for both elongated and four-fold symmetric trap potentials. The change in vortex number between the initial and final states is indicated in parentheses and serves as a measure of the rotation quench strength. The bottom row displays the fluctuation of the number of vortices after each quench, which increases with increase in the magnitude of rotation quenches.

Table I presents the number of vortices in the ground state for each rotation frequency considered in this work, for both the elongated and four-fold symmetric traps. The difference in the vortex numbers between the initial state and each target frequency is also listed, providing an estimate of the rotation quench strength. Notably, for the four-fold symmetric trap, the vortex number remains unchanged between $\omega = 2.2$ and $\omega = 2.15$. However, the vortex positions are more spread out at $\omega = 2.15$, indicating structural changes despite the identical count. We also report the number of vortices during the post-quench dynamics for each case. Interestingly, the fluctuation of the vortex number increases with the magnitude of the quench. Thus, the rotation quench leads to an out-of-equilibrium dynamics that generally involves many excitations and away from the ground state.

II. METHODOLOGY AND NUMERICAL CONVERGENCE

This section provides a brief discussion of the multiconfigurational time-dependent Hartree method for bosons (MCTDHB) and the convergence of the many-body dynamics following the rotational quenches.

A. Methodology

In MCTDHB, the wave-function of the interacting bosons is expanded over a set of permanents, which are the symmetrized bosonic states of N bosons distributed over M single-particle states

$$|\Psi(t)\rangle = \sum_{n}^{N_{\text{conf}}} C_n(t)|n,t\rangle, \tag{2.1}$$

where $n = (n_1, n_2, \ldots, n_M)$ represents the occupation of the orbitals, and each component n_j corresponds to the occupancy of the j-th orbital. The number of possible configurations is $N_{\text{conf}} = \frac{(N+M-1)!}{N!(M-1)!}$. The condition $\sum_{j=1}^{M} n_j = N$ ensures the conservation of the total number of particles. MCTDHB stands out as an efficient method for solving the time-dependent many-body Schrödinger equation [1–3]. The key strength of MCTDHB lies in its ability to dynamically adapt the sampled Hilbert space to the evolving state of the many-body system, thus making it a highly efficient method. Theoretically, the permanents $|n;t\rangle$ form a complete basis as the number of single-particle functions M approaches infinity. In practice, we employ a finite but sufficiently large M, balancing computational feasibility with high accuracy [1]. As the permanents become time-dependent, truncating the basis can provide a high level of accuracy comparable to that of using the same size of a time-independent basis. To solve the time-dependent many-body Schrödinger equation, we compute the time evolution of the coefficients and the orbitals. By applying the variational principle [10], the equations of motion governing both the time-dependent coefficients and orbitals are derived [2, 3]. Finally, the coupled nonlinear integrodifferential equations are solved using the MCTDH-X software [4, 5].

B. Convergence

We assess the convergence of our many-body results using two main quantities. Firstly, we analyze the convergence of the occupations in the natural orbitals and, secondly, the variance of several observables that are highly sensitive to the many-body effect are examined [11, 12]. Specifically, we analyze the position variances ($\frac{1}{N}\Delta^2_{\hat{X},\hat{Y}}(t)$), the momentum variances ($\frac{1}{N}\Delta^2_{\hat{P}_{X,Y}}(t)$), and the angular momentum variance ($\frac{1}{N}\Delta^2_{\hat{L}_Z}(t)$). Following a rotational quench, convergence depends on the amount of energy and angular momentum introduced into

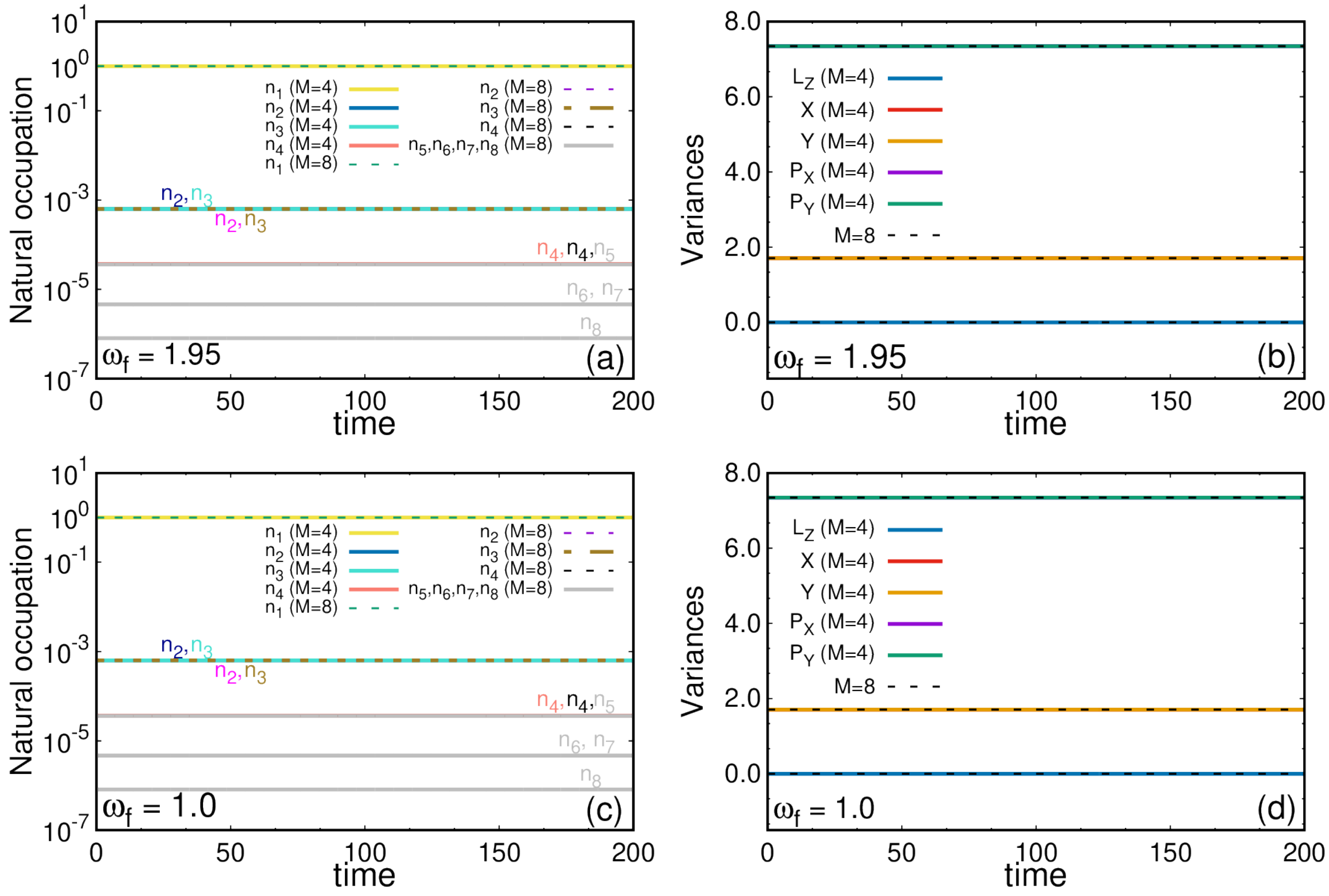

FIG. 2: **Convergence analysis of the natural orbital occupations and the variance of observables in the symmetric trap** (a,c) Evolution of the natural orbital occupations for large and small rotation quenches, computed using $M=4$ and $M=8$ orbitals. During the dynamics, the occupations of the first four natural orbitals from both computations overlap for both quenches, demonstrating a good convergence. (b,d): Time evolution of the angular momentum variance and the position and momentum variances along x- and y-directions are shown for both quenches. Because of the rotational trap symmetry, $\frac{1}{N}\Delta^2_{\hat{X}}(t)$ and $\frac{1}{N}\Delta^2_{\hat{Y}}(t)$, as well as $\frac{1}{N}\Delta^2_{\hat{P}_X}(t)$ and $\frac{1}{N}\Delta^2_{\hat{P}_Y}(t)$ overlaps. The variances are computed using $M=4$ (solid line) and $M=8$ (dotted line) self-consistent orbitals. All quantities shown are dimensionless.

the system, along with the shape of the trapping potential. As time progresses, additional states become engaged in the dynamics, which in turn demands a larger Hilbert space. Obviously, this expansion necessitates a larger number of orbitals to accurately describe the dynamics at longer times.

1. Convergence analysis of the numerical results in the symmetric trap potential

This section exhibits the convergence of the many-body dynamics following the rotation quenches in the symmetric trap. To assess the convergence, all the quantities calculated with $M = 4$ orbitals are compared with $M = 8$ orbitals. Fig. 2(a) illustrates the results of the occupations in natural orbitals (n_i/N) for the large rotation quench. The first four natural orbitals computed with $M = 8$ orbitals align perfectly with those computed using $M = 4$ orbitals. Fig. 2(c) presents the same analysis for the small rotation quench. Similar to the large rotation quench, computations using $M = 8$ orbitals overlap with the results obtained using $M = 4$ natural orbitals. This consistency indicates that the dynamics in the symmetric trap numerically calculated with $M = 4$ orbitals in the main text are well-converged for both the quenches. It is to note that the results for both rotation quenches are identical. Thus, our many-body study reveals that the post-quench state remains highly condensed throughout the time, which explains the strong agreement between the mean-field and the many-body density dynamics (shown in the main text).

Variance serves as a highly sensitive measure, and this sensitivity becomes particularly significant when a small fraction of the system is not in its condensed state. By analyzing the variance of several observables, we gain a comprehensive understanding of how correlations evolve over time after the quenches. Fig. 2(b) and Fig. 2(d) illustrate the time dynamics of variance of different observables for the large and the small rotation quenches, respectively. These include the variance of the angular momentum as well as the position and the momentum variances in both x- and y-directions. No time-dependent behavior is observed across all the variances. Due to symmetry, the variances $\frac{1}{N}\Delta^2_{\hat{X}}(t)$ and $\frac{1}{N}\Delta^2_{\hat{Y}}(t)$ are identical, as are the variances $\frac{1}{N}\Delta^2_{\hat{P}_X}(t)$ and $\frac{1}{N}\Delta^2_{\hat{P}_Y}(t)$. Differences between the mean-field and many-body variance measurements are observed because the mean-field calculations consider the system as a fully condensed state, while the many-body state is not fully 100% condensed, and the deviation stems from the small occupations in the higher orbitals in the many-body calculations. This is not surprising, as it is well documented in the literature [12] that even a single particle out of a million outside the condensate can significantly amplify the discrepancy between mean-field and many-body variances. Regardless of the magnitude of the rotation quench, the eigenfunctions remain unchanged in the symmetric system; thus, the difference between the mean-field and many-body results remains the same throughout the dynamics.

As another check for convergence, the same variances calculated using $M = 8$ orbitals are presented as dotted lines. In all cases, the variances calculated with $M = 8$ time-adaptive orbitals align closely with those obtained using $M = 4$ time-adaptive orbitals, indicating convergence with respect to the number of orbitals. In the symmetric trap, angular momentum is a conserved quantity, resulting in zero angular momentum variance

throughout the dynamics. This characteristic further validates the accuracy of our numerical results.

2. Convergence of quantities in the elongated trap potential

This section discusses the convergence of the many-body results following the rotation quenches in the elongated trap. Due to the broken symmetry along the x-direction, achieving converged results is more challenging in this case compared to the symmetric trap. We address the convergence for small, intermediate, and large rotation quenches separately.

For the small rotation quench, the convergence of the occupations in the natural orbitals is presented in Fig. 3 $(a_1 - a_4)$. We demonstrate the convergence of the first four natural orbitals computed with different numbers of orbitals ($M = 2$, $M = 4$, $M = 8$, and $M = 12$). At short time, all computations using different orbitals are closely aligned. As time progresses, more orbitals are required to accurately describe the state [8]. The computations with $M = 2$ orbitals show a slight divergence from the others, while the $M = 4$ orbital calculations diverge slightly at longer times. But the first four natural orbitals computed using $M = 8$ and $M = 12$ orbitals are overlapped with each other. All other lower natural occupations remain minimal, with $\frac{n_5}{N} \sim \frac{n_6}{N} \sim \frac{n_7}{N} \sim \frac{n_8}{N} \leq 10^{-6}$ throughout the dynamics. In Fig. $4(a_1 - a_5)$, we plot the variance of the angular momentum, the position variance in the x- and y-directions, and the momentum variance in the x- and y-directions, respectively. The results are shown for variance calculations using $M = 2$, $M = 4$, $M = 8$, and $M = 12$ orbitals as well. Calculations with $M = 2$ orbitals show slight deviations, while those with $M = 4$ orbitals exhibit minor divergence in larger time. However, variance calculations with $M = 8$ and $M = 12$ orbitals are in close agreement with each other. These two analyses confirm that the many-body results presented with $M = 8$ orbitals in the main text are fully converged.

The occupations in the natural orbitals for the intermediate rotation quench are presented in Fig. $3(b_1 - b_4)$. We present the first four natural orbitals computed with $M = 2$, $M = 4$, $M = 8$ and $M = 12$ numbers of natural orbitals. Computation with $M = 2$ orbitals starts to deviate after time $t = 20$, indicating that $M = 2$ orbitals are insufficient to describe the dynamics up to time $t = 200$. Similarly, the $M = 4$ orbital computation starts to deviate after $t = 50$, thus failing to provide converged results. However, the first four orbitals computed with $M = 8$ and $M = 12$ orbitals overlap till the time $t = 200$, ensuring the convergence of our many-body results quoted with $M = 8$ in the main text. Fig. $4(b_1 - b_5)$ shows the position variances $(\frac{1}{N}\Delta^2_{\hat{X},\hat{Y}}(t))$, the momentum variances $(\frac{1}{N}\Delta^2_{\hat{P}_{X,Y}}(t))$, and the angular momentum variance $(\frac{1}{N}\Delta^2_{\hat{L}_Z}(t))$, for calculations with $M = 2$, $M = 4$, $M = 8$, and $M = 12$ orbitals as well. While $M = 2$ and $M = 4$ show slight deviations over time, the $M = 8$ and $M = 12$ orbitals yield overlapping results, again confirming the convergence of our main text findings calculated with $M = 8$ orbitals.

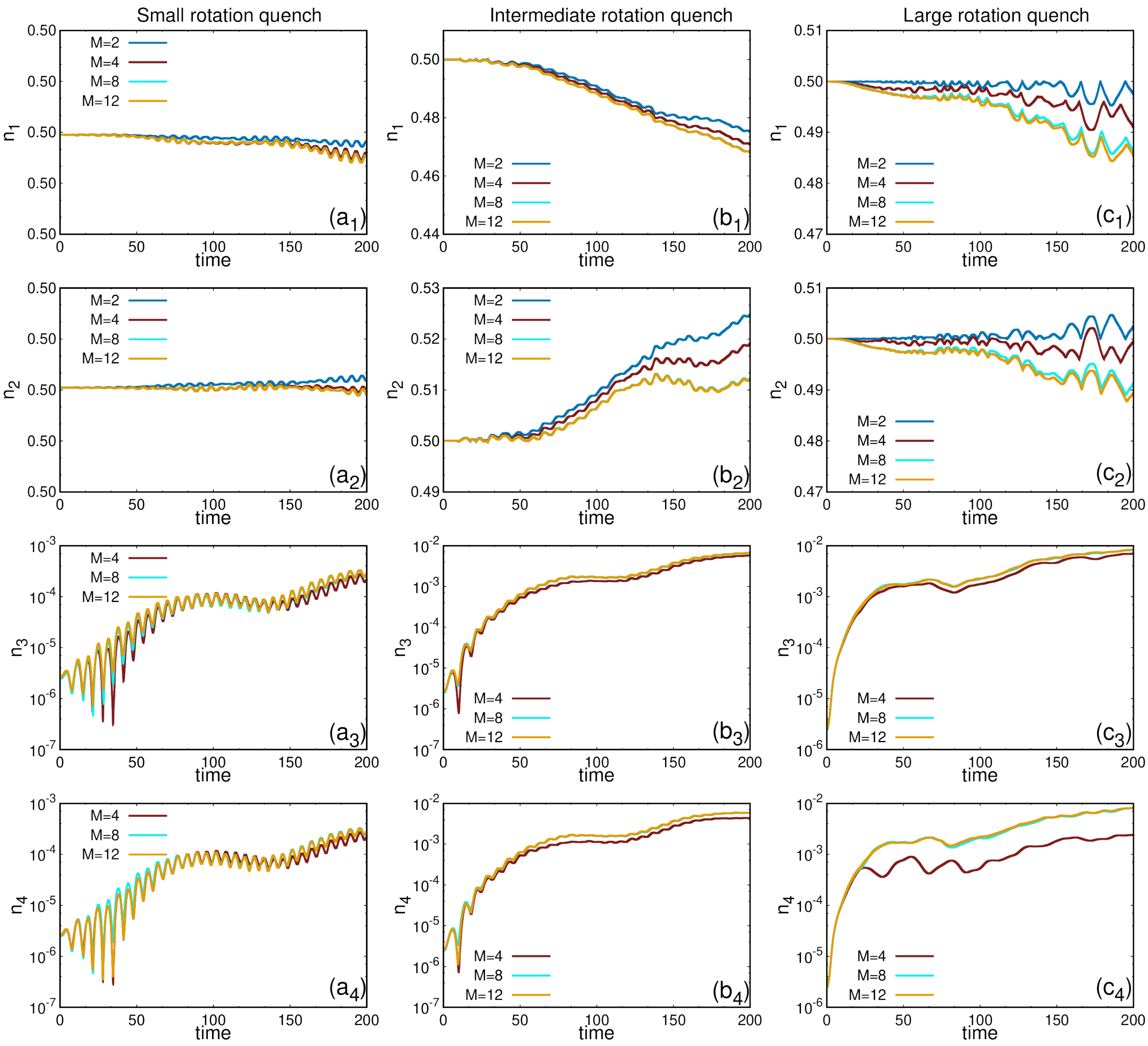

FIG. 3: **Convergence of the natural orbital occupations with increasing number of time-adaptive orbitals in the elongated trap.** The figure illustrates the dynamics of the first four natural orbitals, $\frac{n_{i=1,2,3,4}}{N}$, while varying the number of time-adaptive orbitals in the elongated trap. The analysis is carried out using $M = 2$, 4, 8, and 12 time-adaptive orbitals. (a_1) - (a_4): time evolution of the first four natural orbital occupations for the small rotation quench. (b_1) - (b_4): time evolution of the first four natural orbital occupations for the intermediate rotation quench. (c_1) - (c_4): time evolution of the first four natural orbital occupations for the large rotation quench. This figure highlights that long propagation times require a larger number of self-consistent orbitals to precisely describe the many-body dynamics as compared to short-time dynamics. All quantities shown are dimensionless.

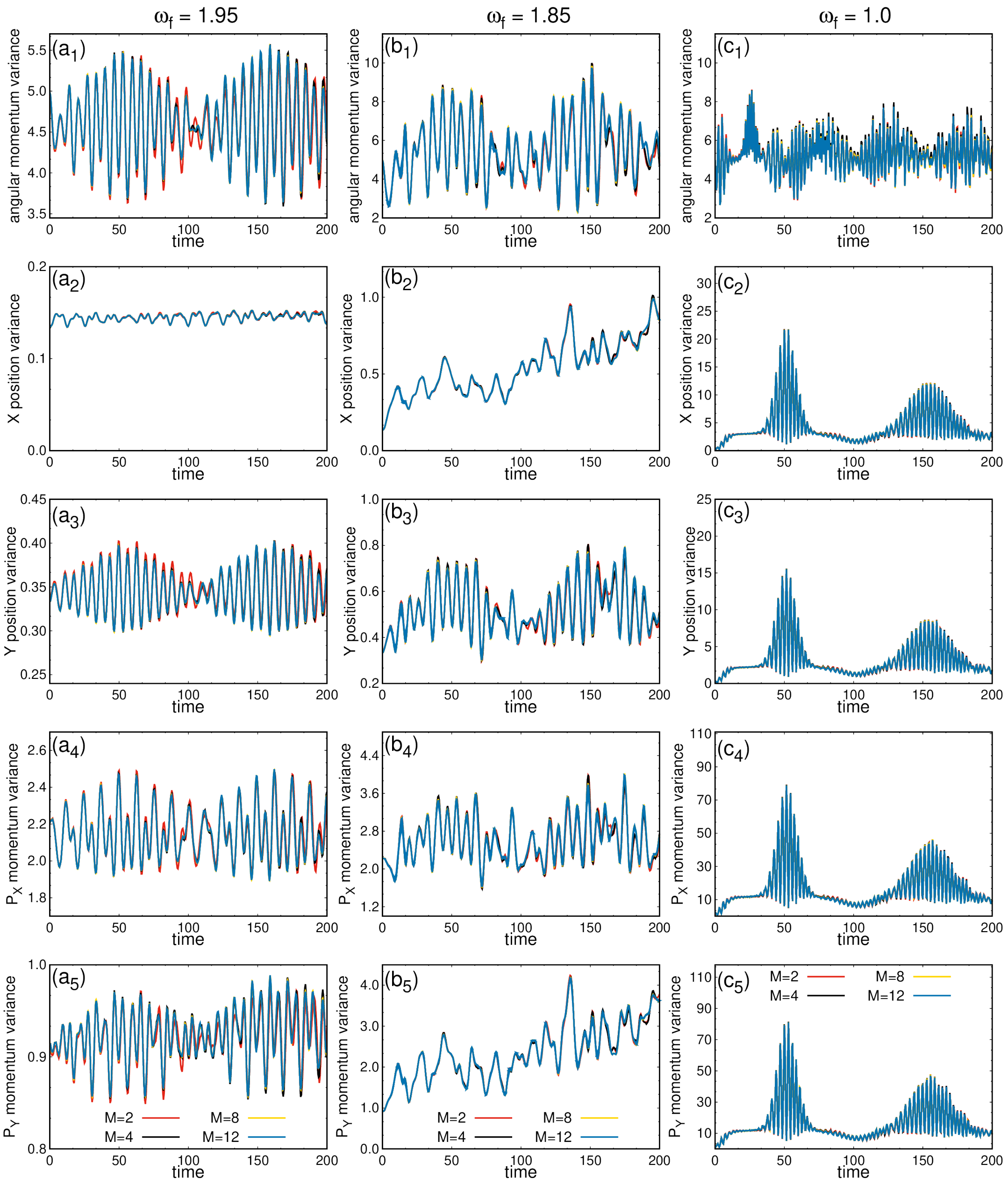

FIG. 4: **Convergence of the variance of different observables with increasing number of time-adaptive orbitals in the elongated trap.** Time evolution of the position, momentum, and the angular momentum variances per particle for different numbers of time-adaptive orbitals ($M = 2$, 4, 8, and 12). Results are shown for small (a_1)-(a_5), intermediate (b_1)-(b_5), and large (c_1)-(c_5) rotation quenches. Each column shows: (1) the angular momentum variance, (2) the position variance in x-direction, (3) the position variance in y-direction, (4) the momentum variance in x-direction, and (5) the momentum variance in y-direction. All quantities shown are dimensionless.

In Fig. 3($c_1 - c_4$), we illustrate the time evolution of the first four natural orbitals for the large rotation quench. The figure clearly illustrates that the $M = 2$ orbital computation is far from convergence. The calculations using $M = 4$ orbitals can describe the short-time dynamics, but fail to capture the long-term dynamics accurately. However, the first four orbitals coincide with the computations using $M = 8$ and $M = 12$ orbitals, confirming the convergence of the main text results with the $M = 8$ orbital calculations. It is worth noting that around $t = 200$, a slight discrepancy is observed between $M = 8$ and $M = 12$ orbitals, emphasizing that for the dynamics beyond $t = 200$, $M = 8$ orbitals are not sufficient. In Fig. 4($c_1 - c_5$), we plot the angular momentum, position, and momentum variances for the large rotation quench computed with $M = 2$, $M = 4$, $M = 8$, and $M = 12$ orbitals. Small deviations are observed in all the variances in $M = 2$ orbitals calculation and a slight deviation in $M = 4$ orbitals calculations at longer times. The results overlap for $M = 8$ and $M = 12$, confirming the reliability of the main findings using $M = 8$ orbitals for the large rotation quench.

3. *Convergence analysis of the measured quantities in the four-fold symmetric trap potential*

In this section, we discuss the many-body results following the rotational quenches in the four-fold symmetric trap computed using different numbers of time-adaptive orbitals, and address the convergence for small and intermediate rotation quenches. For the large rotation quench, converged results are challenging to obtain. To overcome this, we reduce the number of bosons to $N = 4$ and increase the number of orbitals up to $M = 28$. To avoid confusion, we have not provided any result for the large rotation quench with $N = 4$ bosons in the main text. The next section (Sec. II C) focuses solely on the convergence of the measured quantities after the large rotation quench.

The time evolution of the occupations in the natural orbitals after the small rotation quench is presented in Fig. 5($a_1 - a_6$). We demonstrate the convergence of the first six natural orbitals, and the results are obtained with $M = 4$, $M = 8$, $M = 12$, and $M = 16$ orbitals. Initially, the state is four-fold fragmented, with ∼ 25% population in the first four natural orbitals. During the time dynamics, we find that the results with $M = 4$, $M = 8$, $M = 12$, and $M = 16$ orbitals completely overlap for the first four natural orbitals. For the fifth and sixth natural orbitals, the results are compared using $M = 8$, $M = 12$, and $M = 16$ orbitals. The calculations with $M = 12$ and $M = 16$ show slight differences in the fifth and sixth orbitals, but the occupations in these orbitals are very small ($\sim 10^{-5}$). In Fig. 6($a_1 - a_5$), we plot the variance of the angular momentum, position, and momentum operators in the x- and y-directions. The results are shown for calculations using $M = 4$, $M = 8$, $M = 12$, and $M = 16$ orbitals as well. Calculations with $M = 4$ and $M = 8$ orbitals show slight deviations. However, variance calculations with $M = 12$, and $M = 16$ orbitals fall on top of each other. These observations provide confirmation of the convergence of

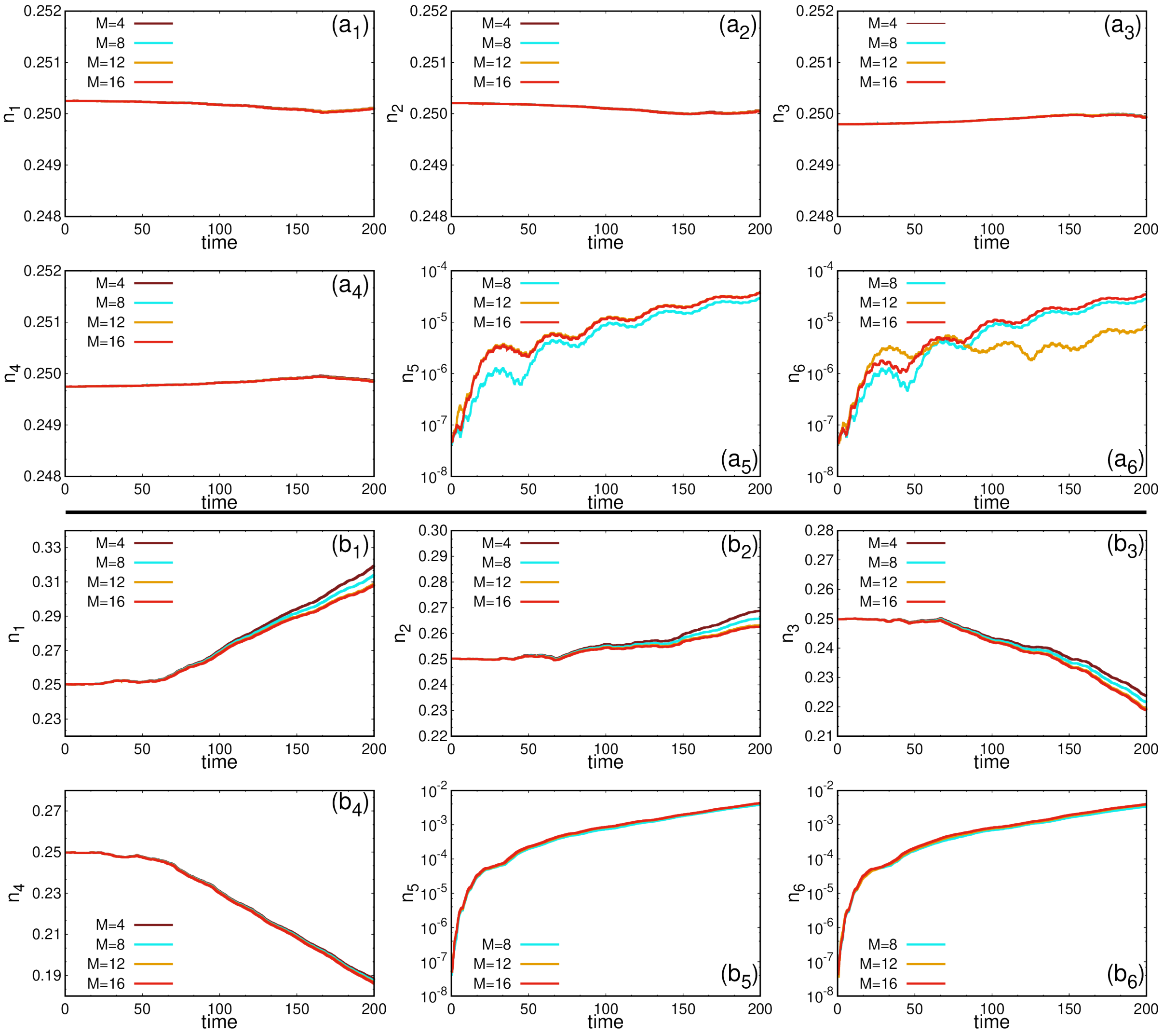

FIG. 5: **Convergence of the natural orbital occupations with increasing number of time-adaptive orbitals in the four-fold symmetric trap.** The convergence of the first six natural orbitals is illustrated. (a_1)-(a_6) show the convergence of the occupations in the natural orbitals for different numbers of time-adaptive orbitals for the small rotation quench. (b_1)-(b_6) display the same for the intermediate rotation quench. We examine the convergence of the first four natural orbitals employing $M = 4$, 8, 12 and 16 time-adaptive orbitals, while the fifth and sixth natural orbitals are analyzed using $M = 8$, 12 and 16 time-adaptive orbitals. All quantities shown are dimensionless.

the results presented with $M = 12$ orbitals in the main text.

The time evolution of the occupations in the natural orbitals for the intermediate rotation quench is presented in Fig. 5$(b_1 - b_6)$. We show the results of the first six natural orbitals computed with $M = 4$, $M = 8$, $M = 12$, and $M = 16$ numbers of natural orbitals. Initially, up to time $t = 50$, all four computations fall on top of each

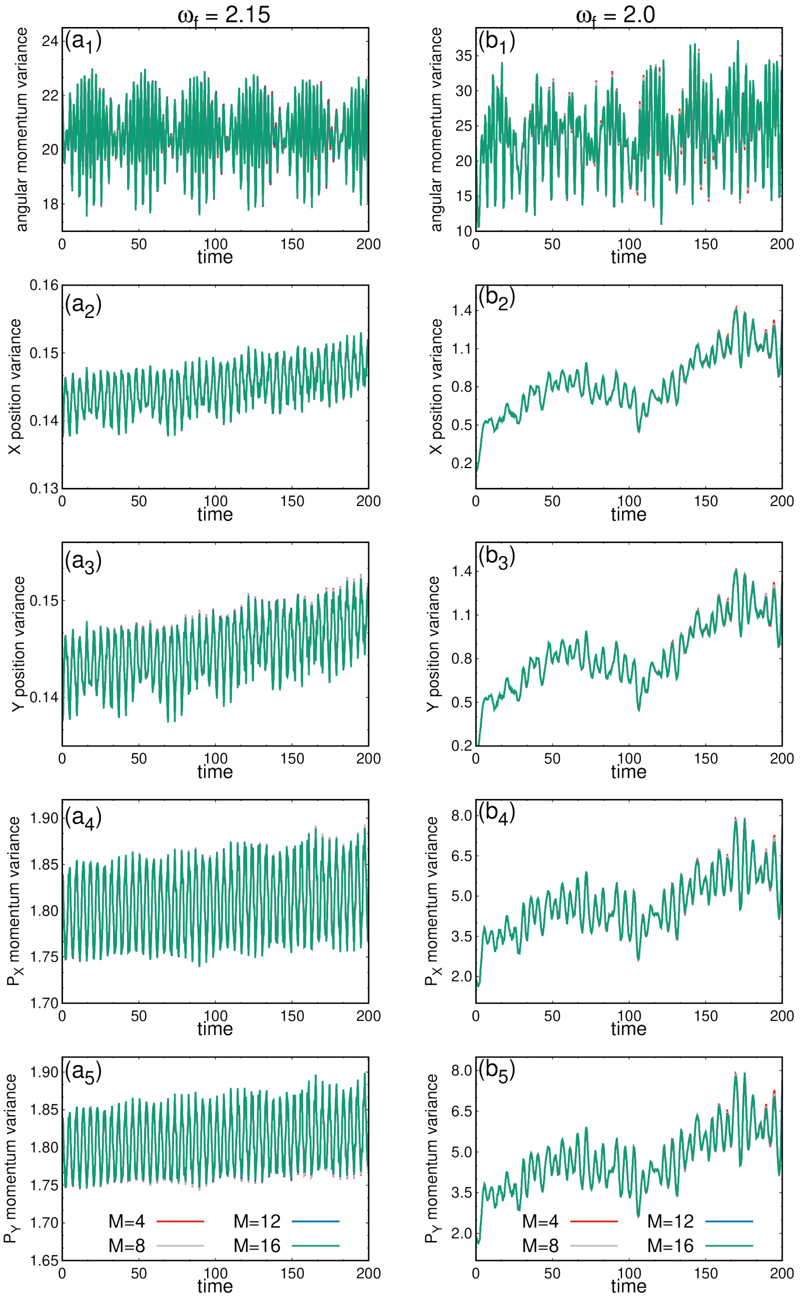

FIG. 6: **Variance of different observables with increasing number of time-adaptive orbitals in the four-fold symmetric trap.** The convergence is analyzed for different number of time-adaptive orbitals ($M = 4$, 8, 12, and 16). Left column ($a_1 - a_5$) shows results for the small rotation quench and the right column ($b_1 - b_5$) for the intermediate rotation quench. Panels display the convergence of: (a_1, b_1) angular momentum variance; (a_2, b_2) position variance along x-direction; (a_3, b_3) position variance along y-direction; (a_4, b_4) momentum variance along x-direction; (a_5, b_5) momentum variance along y-direction. All quantities shown are dimensionless.

other. At later times, the occupations in the natural orbitals calculated using $M = 4$ and $M = 8$ orbitals diverges from $M = 12$ and $M = 16$ orbital calculations. However, the computations for the first six orbitals with $M = 12$ and $M = 16$ orbitals overlap until time $t = 200$, ensuring the convergence of our many-body results quoted with $M = 12$ in the main text. It is noteworthy to mention that although the results are not fully converged with $M = 4$ and $M = 8$ orbitals calculations, they display the same qualitative nature, i.e., $\frac{n_1}{N}$ and $\frac{n_2}{N}$ start to increase from 25%, and $\frac{n_3}{N}$ and $\frac{n_4}{N}$ decrease from the initial 25% occupation. Thus, one can get an idea of the nature of the dynamics from the $M = 4$ and $M = 8$ computations. In Fig. 6($b_1 - b_5$), we plot the position variances ($\frac{1}{N}\Delta^2_{\hat{X},\hat{Y}}(t)$), the momentum variances ($\frac{1}{N}\Delta^2_{\hat{P}_{X,Y}}(t)$), and the angular momentum variance ($\frac{1}{N}\Delta^2_{\hat{L}_Z}(t)$) for the intermediate rotation quench. The results are shown for variance calculations using $M = 4$, $M = 8$, $M = 12$, and $M = 16$ orbitals as well. The calculations with $M = 4$ and $M = 8$ orbitals exhibit slight deviations in longer times. However, the variance calculations with $M = 12$, and $M = 16$ orbitals overlap, which further supports the convergence of the results calculated using $M = 12$ orbitals in the main text.

C. Numerical convergence after the large rotation quench in four-fold symmetric trap

In the four-fold symmetric trap, a large rotation quench represents a significant perturbation to the system, involving a substantial amount of energy and angular momentum being pumped into it. Accurately capturing the dynamics of such a highly excited and complex system requires considering a large number of orbitals in the many-body calculation. However, managing such a large configuration system becomes computationally challenging. To make the problem tractable while retaining the essential physics, we choose to reduce the number of bosons to $N = 4$. By judiciously choosing the small number of particles and employing a very large number of orbitals (up to $M = 28$), we strike a balance between computational feasibility and numerical accuracy. Obviously, for the mean-field calculations, N can be as large as revenant, as the dynamics are controlled solely by the mean-field interaction parameter Λ.

The ground state properties in the four-fold symmetric trap for different rotation frequencies have already been explored in Sec. I. The initial state is prepared with rotation frequency of $\omega_i = 2.2$ and the interaction parameter of $\Lambda = 0.1$. The initial density splits into four sub clouds due to strong rotation, with the ground-state energy per particle of $\frac{E}{N} = -7.96$ and the average angular momentum of $\frac{1}{N}\langle \hat{L}_Z \rangle = 19.49$. The system is quenched to $\omega_f = 1.6$ and the dynamics are explored through the measures of one-body density, occupation in the natural orbitals, the average angular momentum, and the variance of several observables.

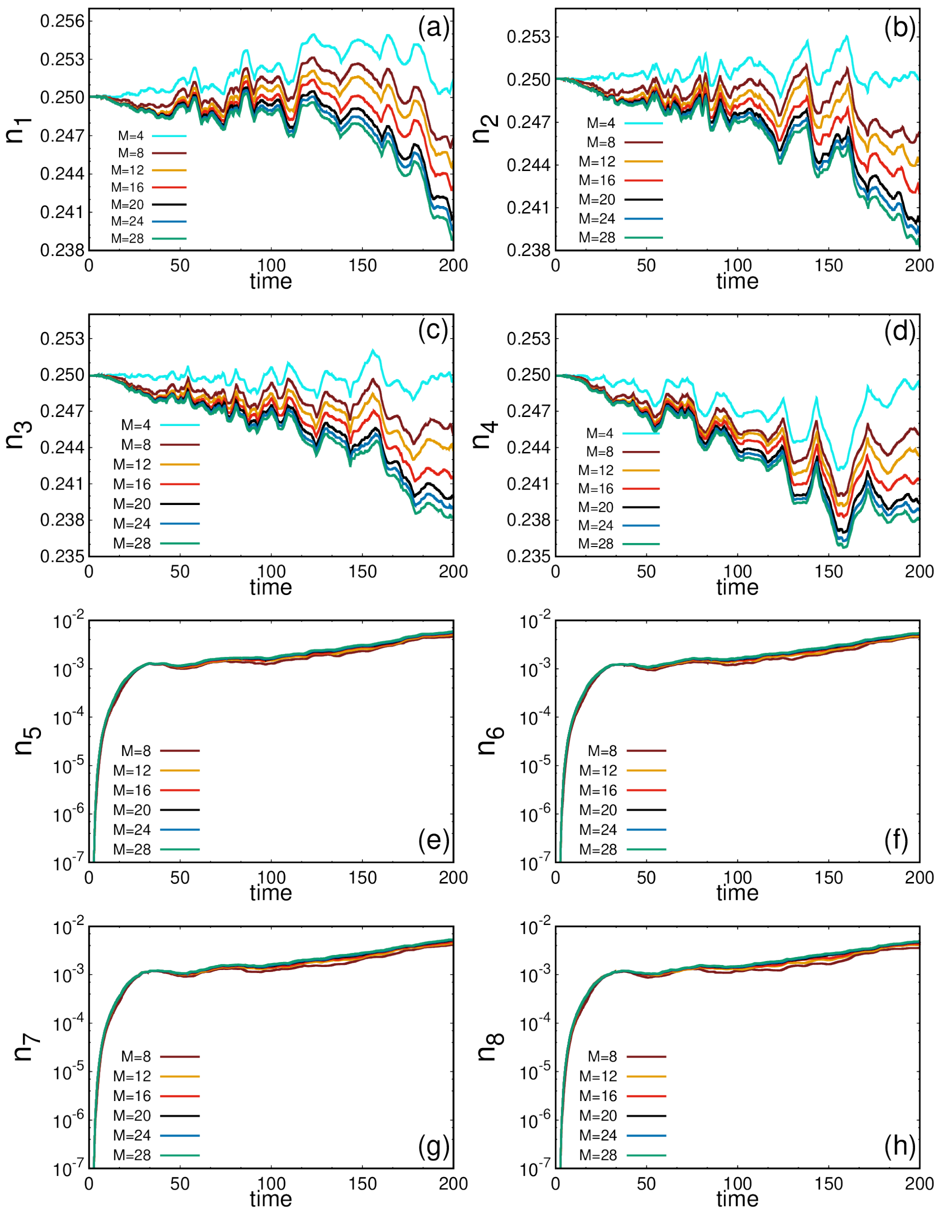

FIG. 7: **Convergence of the natural occupations with increasing number of time-adaptive orbitals for the large rotation quench in the four-fold symmetric trap.** The time evolution of the first eight natural orbitals is depicted. Calculations are conducted for $N = 4$ bosons. The convergence is demonstrated using $M = 4$, 8, 12, 16, 20, 24, and 28 time-adaptive orbitals. All quantities shown are dimensionless.

1. Occupation in the natural orbitals and its numerical convergence

In Fig. 7, we present the time evolution of the first eight natural orbitals, computed using varying numbers of orbitals ($M = 4$, $M = 8$, $M = 12$, $M = 16$, $M = 20$, $M = 24$, and $M = 28$) in the many-body calculation. All the

calculations are plotted on a single graph to illustrate the pace of convergence of the results. The calculations carried out with $M = 4$ and $M = 8$ showcase a significant deviation from the rest within a short time. Nonetheless, the calculations with a high number of orbitals also lack accuracy in describing the dynamics over a long period, as evident in the difference at $t = 200$ for the first four natural orbitals, computed with different orbitals. This analysis reveals two key observations: (i) up to approximately $t = 100$, the outcomes for $M = 24$ and $M = 28$ coincide precisely, indicating that up to $t = 100$, the outcomes from $M = 24$ can be considered as converged. (ii) Fig. 7 clearly illustrates a downward trend for the first four orbitals. The difference between successive computations decreases with increasing orbital number. Thus, we can assert that, while complete convergence is not achieved at the maximum computational time in our calculation, the overall graph pattern remains consistent.

2. Convergence of the average angular momentum and variances

In Fig. 8(a)-(f), we plot the angular momentum, angular momentum variance ($\frac{1}{N}\Delta^2_{\hat{L}_Z}(t)$), position variances ($\frac{1}{N}\Delta^2_{\hat{X},\hat{Y}}(t)$), and the momentum variances ($\frac{1}{N}\Delta^2_{\hat{P}_{X,Y}}(t)$). The variances in the x- and y-directions are identical, both in position and momentum spaces. This is because of the four-fold rotational symmetry of this confinement. To assess the convergence of the calculations, we plot the results using different orbitals ranging from $M = 4$ up to $M = 28$. These results also align with our previous conclusions regarding the convergence of the calculations.

III. ANALYSIS OF THE VORTICES FOR INTERMEDIATE ROTATION QUENCH IN THE ELONGATED AND FOUR-FOLD SYMMETRIC TRAPS

Vortices, which are topological defects in the condensate, arise due to the rotational motion of the BEC and are influenced by factors such as trap geometry and interparticle interactions [13]. For instance, in a ring trap, the vortex configurations are influenced by the interplay between the angular velocity and the interaction strength [14].

In Fig. 9, we plot the density at the specified time as quoted in the main text and the corresponding phases. We calculate the vortices from the phase of the wave-function by identifying the points where the phase circulates by 2π [15]. We analyze the phase structure following the intermediate rotation quench in both the elongated and four-fold symmetric traps. The analysis is performed for mean-field results, as the density dynamics in this regime closely resemble those obtained from the many-body treatment. A comprehensive many-body analysis would require consideration of excited orbitals—specifically, a two-orbital analysis in the elongated trap (due to significant occupation in both orbitals), and at least four orbitals in the four-fold symmetric

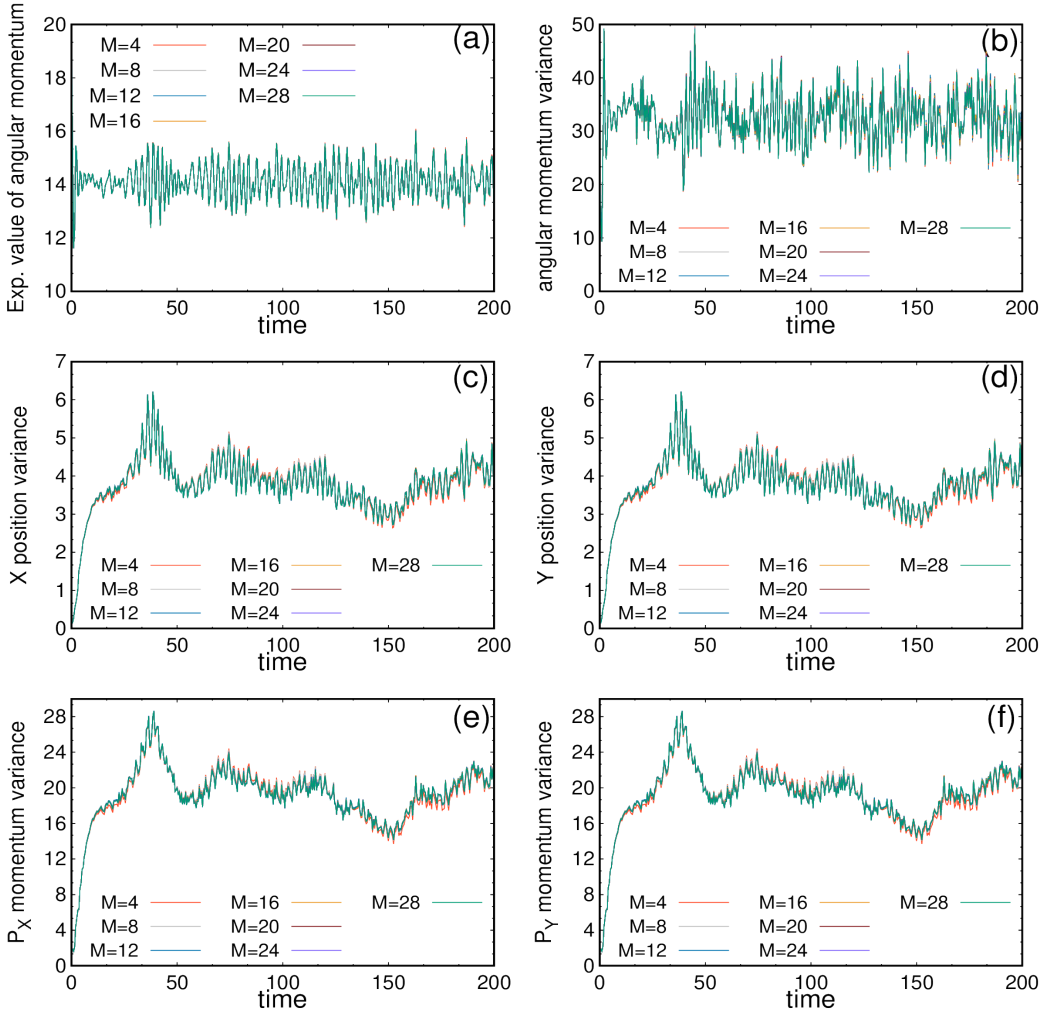

FIG. 8: **Average angular momentum and the variance of different observables for the large rotation quench in the four-fold symmetric trap.** (a) Angular momentum expectation value per particle, $\frac{1}{N}\langle\Psi|\hat{L}_z|\Psi\rangle$, for the system of $N = 4$ bosons is depicted. (b)-(f): Variances of several observables and their convergence with increasing number of time-adaptive orbitals. We demonstrate the convergence of the results using $M = 4$, 8, 12, 16, 20, 24, and 28 time-adaptive orbitals. All quantities shown are dimensionless.

trap—which lies beyond the scope of the present work [16]. Fig. 9(a_1–a_5) shows the density profiles at different times following the quench from $\omega_i = 2.0$ to $\omega_f = 1.85$ in the elongated trap. The corresponding phase distributions are presented in panels (b_1–b_5) of the same figure. Locations where the phase winding number equals a multiple of 2π are indicated by red solid circles, and the same positions are marked with white solid dots in the density plots for reference. This analysis is carried out on a 256×256 spatial grid points. While a lower grid resolution is sufficient to achieve convergence, as discussed earlier, the higher resolution is used here to enhance visual clarity. Notably, a few vortices are present in the density distribution from the early stages of the quench,

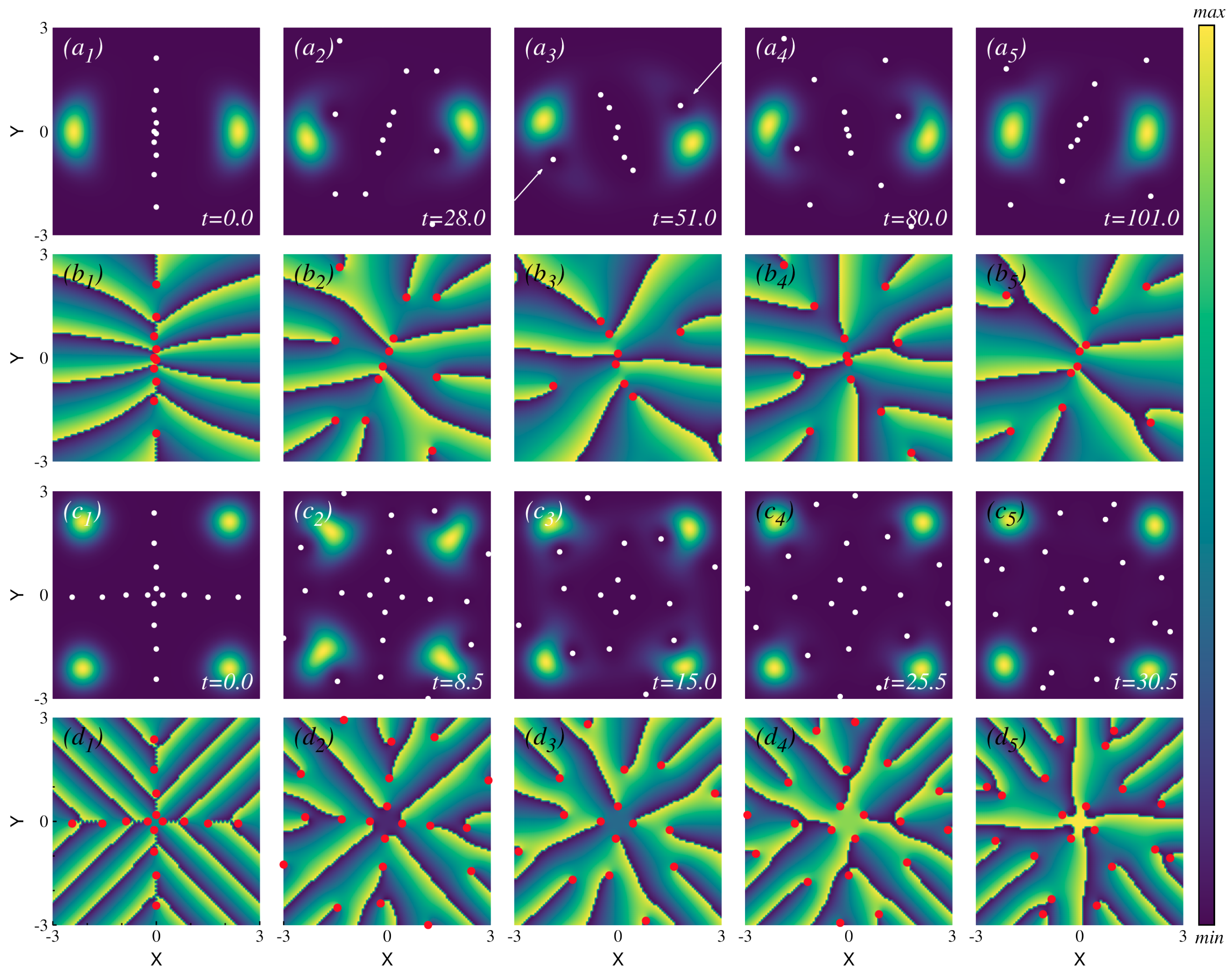

FIG. 9: **Density and phase profiles following an intermediate rotation quench in the elongated and four-fold symmetric traps.** Panels (a_1–a_5) and (c_1–c_5) show the density distributions and the positions of vortices (solid white dots) for quenches in the elongated and four-fold symmetric traps, respectively. The corresponding phase profiles, computed from the mean-field wavefunction, are shown in panels (b_1–b_5) for the elongated trap and (d_1–d_5) for the four-fold symmetric trap. All quantities are presented in dimensionless units.

and their positions evolve with time. Over time, density minima emerge within the two density clouds [see arrows in Fig. 9(a_3)]. This analysis confirms that these density minima correspond to vortices rather than mere density defects, as the vortex positions coincide precisely with the locations of the minima. In some systems, vortices located near the centre of the condensate have higher energy and contribute more significantly to the total angular momentum than those near the periphery [17, 18]. Thus, the angular momentum is determined

not only by the number of vortices but also by their positions, which play a crucial role.

We now focus on analyzing how angular momentum changes in relation to the vortex movement. Following the intermediate rotation quench, we observe that the vortices start to move, with their distances from the center varying over time. This periodic motion leads to oscillations in the angular momentum. Additionally, the change in oscillation amplitude can be attributed to density deformations in regions where vortices are visible within the cloud. For a clearer visualization of this process, a full video of the vortex dynamics and time-dependent angular momentum is provided in the supplementary materials.

A further analysis is conducted for the four-fold symmetric trap. The resulting density profiles are presented in Fig. 9 $(c_1 - c_5)$ with white dots are the positions of the vortices and the phases in Fig. 9 $(d_1 - d_5)$ with the red dot marks are the vortex positions as determined from the phase data. A similar type of dynamics is observed here, where the oscillations arise due to the rearrangement of vortex positions with time. During the dynamics, the vortices become partially visible in Fig. 9(c_2) and Fig. 9(c_4) and more prominently in Fig. 9(c_3) within the high density region. This is responsible for the reduction in the oscillation amplitude of the angular momentum, similarly to the discussion above of the elongated trap. Furthermore, a full vortex dynamics video is available in the supplementary material demonstrating the behavior.

In summary, after the rotation quench in the asymmetric anharmonic traps, the vortices start moving. In the small rotation quench, the vortices move in a repeating pattern (not shown), and hardly no deformation in the density occurs during the dynamics, making the angular momentum to oscillate with a steady amplitude. In the intermediate rotation quench, the relative positions of the vortices with respect to the center change periodically, which drives the oscillations observed in the angular momentum dynamics. Additionally, deformations in the shape and structure of the density clouds manifest as variations in the amplitude of the oscillations. Full videos of the dynamics are enclosed. A deeper, microscopic understanding of the precise role vortices play in contributing to angular momentum during such complex dynamics is a subject for future investigation.

---

[1] A. U. J. Lode, C. Lévêque, L. B. Madsen, A. I. Streltsov and O. E. Alon, Rev. Mod. Phys. **92**, 011001 (2020).

[2] A. I. Streltsov, O. E. Alon and L. S. Cederbaum, Phys. Rev. Lett. **99**, 030402 (2007).

[3] O. E. Alon, A. I. Streltsov and L. S. Cederbaum, Phys. Rev. A **77**, 033613 (2008).

[4] A. U. J. Lode, M. C. Tsatsos, E. Fasshauer, S. E. Weiner, R. Lin, L. Papariello, P. Molignini, C. Lévêque, M. Buettner, J. Xiang, S. Dutta, R. Roy, Y. Bilinskaya, and M. Eder. MCTDH-X: The multiconfigurational time-dependent Hartree method for indistinguishable particles software (2024).

[5] R. Lin, P. Molignini, L. Papariello, M. C. Tsatsos, C. Lévêque, S. E. Weiner, E. Fasshauer, R. Chitra and A. U. J. Lode, Quantum Sci. Technol. **5**, 024004 (2020).

[6] A. U. J. Lode, Phys. Rev. A **93**, 063601 (2016).

[7] E. Fasshauer and A. U. J. Lode, Phys. Rev. A, **93** 033635 (2016).

[8] A. U. J. Lode, K. Sakmann, O. E. Alon and L. S. Cederbaum, A. I. Streltsov, Phys. Rev. A **86**, 063606 (2012).

[9] R. Roy and O. E. Alon, Phys. Rev. A **111**, 043307 (2025).

[10] P. Kramer and M. Saraceno, *Geometry of the Time-Dependent Variational Principle in Quantum Mechanics* (Springer Berlin Heidelberg, Berlin, Heidelberg, 1981).

[11] O. E. Alon, Symmetry, **11** (2019).

[12] S. Klaiman and O. E. Alon, Phys. Rev. A, **91** 063613 (2015).

[13] N.G. Parker, B. Jackson, A.M. Martin and C.S. Adams, Atomic, Optical, and Plasma Physics, **45** Springer, Berlin, Heidelberg (2008).

[14] G. Tomishiyo, L. Madeira and M. A. Caracanhas, Physics of Fluids **36**, 067121 (2024).

[15] A. L. Fetter, Rev. of Mod. Phys., **81**, 647 (2009).

[16] S. E. Weiner, M. C. Tsatsos, L. S. Cederbaum and A. U. J. Lode, Sci. Rep. **7**, 40122 (2017).

[17] T. Yang, Z. Hu, S. Zou, and W. Liu, Sci. Rep. **6**, 29066 (2016).

[18] A. A. Svidzinsky and A. L. Fetter, Phys. Rev. A **62**, 063617 (2000).
